# Role of Bile Acids in Bariatric Surgery

**DOI:** 10.3389/fphys.2019.00374

**Published:** 2019-04-02

**Authors:** Wenting Wang, Zhiqiang Cheng, Yanlei Wang, Yong Dai, Xiang Zhang, Sanyuan Hu

**Affiliations:** ^1^Department of Obstetrics and Gynecology, The Second Hospital of Shandong University, Jinan, China; ^2^Department of General Surgery, Qilu Hospital of Shandong University, Jinan, China

**Keywords:** bariatric surgery, bile acids, metabolic disorders, diabetes type 2, gut microbiota

## Abstract

Bariatric surgery has been proved to be effective and sustainable in the long-term weight-loss and remission of metabolic disorders. However, the underlying mechanisms are still far from fully elucidated. After bariatric surgery, the gastrointestinal tract is manipulated, either anatomically or functionally, leading to changed bile acid metabolism. Accumulating evidence has shown that bile acids play a role in metabolic regulation as signaling molecules other than digestive juice. And most of the metabolism-beneficial effects are mediated through nuclear receptor FXR and membrane receptor TGR5, as well as reciprocal influence on gut microbiota. Bile diversion procedure is also performed on animals to recapitulate the benefits of bariatric surgery. It appears that bile acid alteration is an important component of bariatric surgery, and represents a promising target for the management of metabolic disorders.

## Introduction

Bariatric surgery has been proved to be both effective and sustainable in the long-term weight-loss and remission of metabolic disorders (Adams et al., 2017; Schauer et al., 2017). However, the underlying mechanisms are still far from fully elucidated. One interesting phenomenon observed after bariatric surgery is the increase of serum total bile acids. Actually, apart from the purely restrictive procedure, adjustable gastric banding (AGB) (Kohli et al., 2013a), it has been reported that all other types of bariatric surgery are associated with postsurgical increase of serum total bile acids (Steinert et al., 2013; Albaugh et al., 2015; Ferrannini et al., 2015; Han et al., 2015). And this phenomenon has raised concern about the relationships between bile acids and bariatric surgery. Bile acids are known as the “intestinal soap,” for their physiological effect on facilitating dietary fat and fat-soluble vitamin absorption. It is not until the late 1990s that bile acids had been found taking effect as signaling molecules (Makishima et al., 1999). Most of the metabolic benefits of bile acids are mediated through nuclear or membrane receptors, or interaction with gut microbiota. In the present review, we focus on two major questions: (i) why serum bile acids are increased after bariatric surgery? (ii) how the altered bile acid metabolism contributes to the metabolic benefits of bariatric surgery?

## Mechanisms of Serum Bile Acid Elevation After Bariatric Surgery

Bile acids are synthesized from cholesterols in the liver, stored in the gallbladder and expelled into the duodenum in response to enteral stimuli. Approximately, 95% of luminal bile acids are recycled into the portal vein in the terminal ileum and reutilized by the liver, forming the “enterohepatic circulation.” Serum bile acids represent the excess bile acids that cannot be recycled by the liver during the enterohepatic circulation and thus present in the peripheral circulation. Increased serum bile acids may derive from increased synthesis in the liver, more reabsorption in the ileum and/or decreased excretion in the feces.

In Roux-en-Y gastric bypass (RYGB) and biliopancreatic diversion (BPD), shortened route of the enterohepatic circulation (the biliopancreatic limb and the common limb) expedites the contact of luminal bile acids with the ileum, a major area for bile acid reabsorption, leading to earlier and more active bile acid reabsorption (Ferrannini et al., 2015). This explanation is also supported by an experimental procedure performed on rats termed ileal interposition (IT), in which the interposition of a segment of the ileum into the proximal jejunum shows greater bile acid reabsorption and increased serum bile acids (Cummings et al., 2013). It has been speculated that the length ratio of the biliopancreatic limb and the common limb represent a major determinant of postoperative serum bile acid concentrations. Postoperative serum bile acid concentrations are likely to be greater with longer biliopancreatic limb and shorter common limb, as evidenced by both animal (Han et al., 2015; Miyachi et al., 2016) and human studies (Mika et al., 2018). The precise mechanism has yet been elucidated. In BPD, the functional common limb left is less than one meter (Tacchino et al., 2003), and the increased luminal bile acid reabsorption cannot compensate for substantial fecal bile acid loss. Therefore, the increased hepatic bile acid synthesis also contributes to increased serum bile acids as a complementary mechanism (Scopinaro, 2006; Ferrannini et al., 2015). Compared to BPD, RYGB as well as duodenal-jejunal bypass (DJB, an experimental procedure designed to investigate the mechanism of diabetes remission with no effect on weight-loss), has enough intestinal bile acid reabsorption area, with either decreased (de Siqueira Cardinelli et al., 2019) or unchanged fecal bile acid excretion (Li et al., 2011; Bhutta et al., 2015), and the hepatic bile acid synthesis is decreased (Ferrannini et al., 2015; Zhang et al., 2016), probably due to a feedback regulation in response to the increased serum bile acids.

For vertical sleeve gastrectomy (VSG), only limited data are available regarding postsurgical serum bile acids. One human study showed immediate increase of serum bile acids after surgery (Kindel et al., 2018), while two other human studies showed unchanged serum bile acids until 1 or 2 years after surgery, with only statistically non-significant increase (Nakatani et al., 2009; Haluzikova et al., 2013). In contrast, animal studies showed consistently increased serum bile acids after VSG (Cummings et al., 2012; Myronovych et al., 2014a,b). Due to the lack of direct anatomical manipulation of the gut, the increased serum bile acids after VSG are likely to be a secondary effect of altered gastrointestinal function (Sips et al., 2018) and progressively intestinal adaption. It has been reported that VSG accelerates gastric emptying and intestinal motility (Mans et al., 2015). And Myronovych et al. (2014b) has found decreased hepatic bile acid synthesis, increased intestinal villus length and enlarged bile acid reabsorption intestinal area, which might explain the increased serum bile acids after VSG.

## Bile Acids, Fxr and Bariatric Surgery

### Role of FXR in Maintaining Bile Acid Homeostasis

The farnesoid X receptor (FXR, NR1H4), an orphan member of the nuclear receptor family, was first identified as a natural receptor for bile acids in 1999 (Makishima et al., 1999; Parks et al., 1999; Wang et al., 1999). Subsequently, bile acids were found transcriptionally regulating their own synthesis and enterohepatic transport by repressing CYP7A1 (a rate-limiting enzyme for bile acid synthesis in the liver) via hepatic FXR-SHP-CYP7A1 pathway (Goodwin et al., 2000; Lu et al., 2000). Later, fibroblast growth factor 19 (FGF19, in rodents ortholog FGF15), secreted mainly from the ileum in response to intestinal FXR activation, was found as an enterohepatic signal to regulate bile acid homeostasis as well (Inagaki et al., 2005). Circulating FGF15/19 within the portal venous system targets FGF receptor 4 (FGFR4) in the liver, and inhibits CYP7A1 expression and bile acid synthesis, thus working as components of a gut-liver signaling pathway that synergizes with hepatic FXR-SHP-CYP7A1 pathway to regulate bile acid homeostasis (Inagaki et al., 2005).

### Role of FXR in Metabolism Regulation

The metabolism-regulating effect of FXR is more complex compared to bile acid homeostasis maintenance. A variety of genetic and pharmacological animal models have been established to investigate the role of FXR signaling in body weight management and metabolism regulation. Whole body FXR knock-out mice (*FXR^-/-^*) displayed impaired glucose disposal and elevated blood glucose concentrations, as a result of blunted insulin pathway in the liver, peripheral adipose tissue and skeletal muscles, although FXR is not expressed in skeletal muscles (Cariou et al., 2006; Ma et al., 2006). Meantime, plasma triglycerides, cholesterols and free fatty acids were elevated, with severe liver fat accumulation, however, surprisingly, this kind of mouse model was protected from diet- or genetically-induced obesity, probably because the adipocyte differentiation was impaired in the absence of FXR (Sinal et al., 2000; Cariou et al., 2006). In contrast, supplementation of whole-body FXR agonist (GW4064) in *ob/ob* mice has multiple beneficial effects, indicating that FXR could be a potential therapeutic target for metabolic disorders and bariatric surgery (Cariou et al., 2006).

Since FXR is abundant in the liver and small intestine, some studies concentrate on its tissue-specific signaling. In the liver, FXR activation lowers plasma triglycerides via FXR-SHP-(SREBP-1c) pathway (Watanabe et al., 2004), and improves hyperglycemia by inhibiting gluconeogenesis and improving insulin sensitivity (Zhang et al., 2006). In contrast, liver-specific *FXR^-/-^* mice showed increased plasma triglycerides, and unlike whole-body FXR knock-out, were not protected from diet-induced obesity and insulin resistance (Watanabe et al., 2004). In the small intestine, the physiological role of FXR is primarily mediated by FGF15/19 whose metabolism-beneficial functions resemble those of insulin in stimulating protein and glycogen synthesis and inhibiting gluconeogenesis, but does not lead to body weight gain or hepatic fat accumulation (Kir et al., 2011).

### Bile Acid-FXR Pathway in Bariatric Surgery

Since bile acids are natural ligands for FXR, it has been believed that bile acid-FXR pathway contribute to the beneficial effects of bariatric surgery. However, no consensus has been reached so far. The essential role of FXR in bariatric surgery was first demonstrated by a milestone research published in 2014. The ability of VSG to reduce body weight and improve glucose tolerance was substantially reduced in the state of genetic disruption of FXR (Ryan et al., 2014). Nevertheless, the results were later questioned by the authors themselves, as the whole body FXR knock-out mice *per se* were protected from diet-induced obesity as well as glucose intolerance, and the observational window might not be long enough (Bozadjieva et al., 2018). Bariatric surgery alters bile acid drainage, either anatomically or functionally, and changes luminal bile acid concentrations and composition. Activation of intestinal FXR by bile acids increases FGF19 following bariatric surgery in humans and represents one mechanism accounting for postsurgical metabolic improvement (Jansen et al., 2011; Pournaras et al., 2012; Gerhard et al., 2013; Haluzikova et al., 2013; Jorgensen et al., 2015; Sachdev et al., 2016). One human study did not support the contribution of FGF19 to the benefit of RYGB, as the increase of FGF19 did not parallel the improvement of glucose tolerance (Jorgensen et al., 2015). And animal studies have shown that both intestine-specific FXR agonist (Fexaramine) (Fang et al., 2015) and antagonist (Glycine-muricholic acid) (Jiang et al., 2015) have the capacity of reducing body weight and improving metabolism. Interestingly, alteration of luminal bile acids after bariatric surgery did not guarantee activation of intestinal FXR. On the contrary, intestinal FXR was intact or even repressed (Kohli et al., 2013b; Goncalves et al., 2015). These unexpected phenomena have been thought closely related to the intestinal bile acid milieu, as the physiological effect of each individual bile acid differs in interacting with FXR, with chenodeoxycholic acid (CDCA) being a potent agonist, deoxycholic acid (DCA) being a moderate agonist (Parks et al., 1999), yet ursodeoxycholic acid (UDCA) (Mueller et al., 2015), and muricholic acid (MCA, only in rodents) being antagonists (Jiang et al., 2015). Surprisingly, intestinal FXR repression also leads to metabolic benefits. One study reported that FXR repression within intestinal L cells led to glucagon-like peptide 1 (GLP-1) secretion hence improving glucose metabolism (Trabelsi et al., 2015). This finding may also partly explain the paradox between intestinal bile supplementation and bile deprivation (e.g., bile sequestrant), both of which have been confirmed effective in enhancing GLP-1 secretion (Trabelsi et al., 2015; Zhang et al., 2016).

It should be noted that most of the studies identifying intestinal FXR repression as a positive metabolic regulator are from animal models, suggesting that the gut may respond differently to FXR-specific targeting between animals and humans.

## Bile Acids, Tgr5, and Bariatric Surgery

G protein coupled receptor 1, also known as TGR5 (GPR131), was discovered as a membrane-type receptor for bile acids in 2002 (Maruyama et al., 2002) and has been confirmed as another essential receptor in maintaining glucose homeostasis after sleeve gastrectomy (McGavigan et al., 2017). However, one latest report revealed that the metabolic benefits were still maintained in *Tgr5^-/-^*, but not *Fxr*^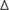/*E*^ mice after a bariatric procedure, suggesting a less important role for TGR5 as opposed to intestinal FXR (Albaugh et al., 2018). Unlike FXR, each individual bile acid is capable of activating TGR5, only with different potency (conjugated form > unconjugated form, LCA > DCA > CDCA > CA) (Maruyama et al., 2002). Therefore, after bariatric surgery, the increased luminal or serum bile acids are able to elicit TGR5-mediated metabolic benefits, and appear to make a complementary contribution to the metabolic benefits of bariatric surgery.

### Luminal Bile Acids, TGR5, and Incretins

In the distal small intestine, TGR5 activation within enteroendocrine L cells in response to luminal bile acids have been confirmed as a critical signaling pathway in promoting GLP-1 secretion in both animal models (Thomas et al., 2009) and humans (Wu et al., 2013). Following bariatric surgery, as a result of gut manipulation and increased luminal bile acids, serum GLP-1 has been found ubiquitously elevated, either acutely (le Roux et al., 2007) or gradually (Borg et al., 2006), and is now referred to as an important, albeit not exclusive, mechanism of bariatric surgery in improving glucose tolerance (Madsbad and Holst, 2014). GLP-1 and glucose-dependent insulinotropic polypeptide (GIP) are known as “incretin hormones” as they mediate the incretin effect, defined as the increased stimulation of insulin secretion elicited by oral compared with intravenous administration of glucose under similar plasma glucose levels (Wu et al., 2016). In health, the incretin effect accounts for 70% of postprandial insulin secretion after oral ingestion of glucose in a glucose-dependent fashion (Nauck et al., 1986). In T2DM, the incretin effect is markedly impaired as a result of the loss of insulinotropic effect of GIP, while GLP-1 still maintains its pleiotropic physiological functions including stimulating insulin secretion, improving hepatic insulin sensitivity and suppressing appetite (Wu et al., 2014, 2016), which might account for the partially restored incretin effect after bariatric surgery (Laferrere et al., 2007). A latest “proof of concept” study has proved that the distal, as opposed to proximal, small intestine is superior in modulating postprandial glucose metabolism in both health and T2DM, of which GLP-1 plays a major role (Zhang et al., 2019). Currently, GLP-1 analogs as well as dipeptidyl peptidase-4 (DDP4, an enzyme for degradation of incretins) inhibitors have been commercialized for the management of T2DM.

### Serum Bile Acids, TGR5, and Energy Expenditure

In the skeletal muscle and brown adipose, increased serum bile acids have the capacity of converting tetraiodothyronine (T4) to triiodothyronine (T3) through TGR5, leading to more energy expenditure, which might facilitate weight loss (Kohli et al., 2013a; Broeders et al., 2015; Watanabe et al., 2006). However, the relevance between TGR5-mediated energy expenditure and bariatric surgery has not been clarified. In a human study, increased skeletal gene expressions of TGR5 downstream targets (mitochondrial COX IV and Kir6.2) were detected after RYGB, but no correlation with resting energy expenditure was found (Kohli et al., 2013a). In addition, the majority of studies suggest decreased energy expenditure after bariatric surgery, which might be a secondary effect of weight loss (Benedetti et al., 2000; Carrasco et al., 2007). In contrast, increased weight-adjusted energy expenditure was also present in some studies (Faria et al., 2012; Rabl et al., 2014). As energy metabolism is related to many factors, such as body composition, exercise or hormones, whether TGR5-mediated energy regulation plays a role in weight-loss after bariatric surgery still warrants further research.

## Microbiota and Bile Acids

### Role of Gut Microbiota in Metabolic Disorders

Gut microbiota, also named as “The Second Genome,” consists of more than 10^14^ microorganisms, most of which have not been fully investigated (Turnbaugh et al., 2007). Five phyla dominate human gut community, including Actinobacteria, Bacteroidetes, Firmicutes, Proteobacteria, and Verrucomicrobia, of which Bacteroidetes and Firmicutes account for over 90% of the whole gut microbiota (Tilg and Kaser, 2011). Along the whole gut, microbiota is not uniformly distributed. Both the abundance and diversity are increased from the proximal to distal gut (Prakash et al., 2011). In the stomach, only bacteria resistant to acidic environment survive (e.g., *Lactobacillus* or *Streptococcus* or *Veillonella*) (Dicksved et al., 2009), while in the large intestine, where the intrinsic environment is suitable for bacterial growth, lives most of the gut bacteria (Prakash et al., 2011). Gut microbiota begins to colonize since newborns, and is related to certain pathological states in adulthood. An identical twin study showed that different physiologic states (obese versus lean) are associated with distinct gut microbiota, despite the same genetic background (Turnbaugh et al., 2009). In obesity, the majority of animal and human studies have demonstrated that the diversity of gut microbiota is decreased, and Phylum Firmicutes is increased at the expense of Phylum Bacteroidetes, independent of food consumption (Ley et al., 2006; Turnbaugh et al., 2006, 2009). In T2DM, inconsistent results have been reported. Larsen et al. found that the proportions of Phylum Firmicutes and Class Clostridia were significantly reduced in T2DM, and Bacteroidetes/Firmicutes ratio was correlated positively and significantly with plasma glucose concentrations (Larsen et al., 2010). Qin et al. used a deep shotgun sequencing method analyzing gut microbial DNA from 345 Chinese individuals, and found that type 2 patients had a moderate degree of gut bacterial dysbiosis, a decline in butyrate-producing bacteria and an increase in diverse opportunistic pathological bacteria (Qin et al., 2012). They also pointed out that compared to inflammatory bowel disease (IBD), the degree of gut microbiota changes in T2DM was not that substantial. Since gut microbiota is influenced by various factors such as life style, dietary, disease, medication, and surgery, the currently available results should be interpreted with caution, and may not be used as a marker for T2DM monitoring at the moment.

### Changes of Gut Microbiota After Bariatric Surgery

Bile acids and gut microbiota are reciprocally affected and are both altered by bariatric surgery. Within the enterohepatic circulation, bile acids in the distal ileum and colon undergo hydrolysis (deconjugation) and subsequent transformation into secondary bile acids. These processes require the catalysis of bile salt hydrolases (BSHs, produced by certain gut bacteria) and the bacterial activity of 7α-dehydroxylation (Ridlon et al., 2006). Meantime, both gut bile acid concentrations and compositions have direct influence on gut microbiota. Increased luminal bile acid concentrations have anti-bacterial effects, killing certain strains of bacterium and make some other bacteria (e.g., *Bilophila wadsworthia*) thrive (Noh and Gilliland, 1993; Devkota et al., 2012). Furthermore, some individual bile acids are capable of activating enteral FXR, thus leading to enteroprotection by promoting related gene expressions and inhibits bacterial overgrowth and mucosal injury in the ileum. All these effects indicate the existence of indirect influence on gut microbiota by gut bile acids (Inagaki et al., 2006). Following bariatric surgery, gut microbiota is changed. In RYGB, at the phylum level, Proteobacteria has been reported to be increased at the expense of Firmicutes (Zhang et al., 2009; Tremaroli et al., 2015), while Bacteroidetes has been found inconsistently changed, either increased (Furet et al., 2010) or decreased (Li et al., 2011; Graessler et al., 2013). In VSG, both animal and human studies showed increased Bacteroidetes and decreased Firmicutes, and rebuilt of the gut microbiota diversity (Jahansouz et al., 2017; Murphy et al., 2017; Shao et al., 2018). At the species level, the outcomes have been inconsistently reported across different studies, further increasing the difficulty of gut microbiota research. As to the interaction of bile acids and gut microbiota after bariatric surgery, it’s similar to an old question: “which came first, the chicken or the egg?” We do not have direct and strong evidence at the moment, but assume that the instantly altered luminal bile acids following bariatric surgery might be the driving force, and the final phenotype of luminal bile acids and gut microbiota is a result of gradual interaction and adaption of each other.

### Gut Microbiota After Bariatric Surgery: A Signal Carrier?

Another question to be answered is how gut microbiota affects the host after bariatric surgery. In fact, the contribution of gut microbiota to the metabolic benefits after bariatric surgery has not been clarified. Based on currently available reports, gut microbiota is likely to be a signal carrier and transmits signals that are able to play a role even in a different host. Direct evidence comes from fecal microbiota transplantation (FMT). Patients with metabolic syndrome experienced significantly increased insulin sensitivity after receiving gut microbiota from lean donors (Vrieze et al., 2012). However, the improvement of insulin sensitivity disappeared at 12 weeks after FMT, suggesting that the beneficial effect of FMT might not last in absence of sustainable exogenous stimulations. Metabolic benefits were also observed in FMT from bariatric surgery recipient donors to mice (Tremaroli et al., 2015), but we do not know whether the effect of bariatric surgery could be recapitulated by FMT in human recipient as well.

Due to technical reasons, it’s difficult to observe the dynamic change of luminal bile acids and gut microbiota frequently after bariatric surgery. And a Dutch population-based cohort showed that the influence of currently realized exogenous and intrinsic host factors could only explain 18.7% of the inter-individual variation of microbial composition (Zhernakova et al., 2016). Therefore, the knowledge of gut microbiota is far from enough and warrants future research.

## Bile Diversion Procedures

In order to isolate the contribution of bile acids to the metabolic benefits of bariatric surgery, some studies have tried to manipulate bile flow directly without altering the gastrointestinal tract (Table 1). Bile diversion (BD) procedure in rats was performed as early as the 1980s, even before the discovery of GLP-1, to investigate the influence of re-routing of bile flow on glucose homeostasis. This early study showed 10% less weight of rats undergoing BD than control, decreased fasting blood glucose and improved glucose tolerance, with no effect on insulin secretion (Manfredini et al., 1985). Another study in 1991 using streptozocin (STZ) -induced diabetic rats showed similar results, except an immediate return of insulin to normal level after surgery (Ermini et al., 1991). It is not until 2013 that the BD procedure had regained attention. Subsequent studies have demonstrated comparable effects of BD with gastric bypass procedure in glucose control in normal rats (Goncalves et al., 2015), diet-induced obese rats (Kohli et al., 2013b), and mice (Flynn et al., 2015). However, under diabetic state, the effects of BD were attenuated compared to DJB, with limited glucose-lowering effect observed. Therefore, to our knowledge, bile acid manipulation appears to be capable of recapitulating the effects of bariatric surgery only in non-diabetic state.

**Table 1:** Bile diversion procedures.

Year	Author	Journal	Model	Diversion site	Weight	Fasting glucose	Glucose tolerance	Insulin	GLP-1
1985	Manfredini G	Am J Physiol	Wistar rats	Second jejunal loop	↓ (week 1)	↓ (week 1)	↓ (week 1)	—	N/A
1991	Ermini M	Acta Diabetol Lat	STZ-induced diabetic rats	Second jejunal loop	↓ (instantly)	↓ (instantly)	N/A	↓ (instantly)	N/A
2013	Kohli R	Endocrinology	Long-Evans obese rats	15, 30 cm distal to Treitz ligament	— (week 5)	↓ (week 5)	↓ (week 5)	↓ (week 4)	↑ (week4)
2015	Flynn CR	Nat Commun	Diet-induced obese mice	Ileum	↓ (week 8)	↓ (week 4)	↓ (week 2)	↓ (week 8)	N/A
2015	Goncalves D	Ann Surg	Sprague-Dawley rats	Mid-jejunum and mid-ileum	— (day 9)	— (day 10)	↓ (day 10)	↑ (day 10)	N/A
2015	Zhang X	Obes Surg	STZ-induced diabetic rats	25 cm distal to Treitz ligament	↓ (week 8)	↓ (week 2)	↓ (week 8)	—	↑ (week8)


## Conclusion

Bile acids play a role in metabolic regulation through various pathways. Alteration of bile acid metabolism is an important component of bariatric surgery, and represents a promising target for the management of metabolic disorders.

## Author Contributions

WW was involved in literature searching and writing of the manuscript. ZC, YW, YD, XZ, and SH were involved in conception and assist in writing. XZ was involved in conception, design, and coordination of the work. All authors critically reviewed the manuscript and have approved the publication of this final version of the manuscript. XZ and SH are the guarantors of this work.

## Conflict of Interest Statement

The authors declare that the research was conducted in the absence of any commercial or financial relationships that could be construed as a potential conflict of interest. The handling Editor declared a past supervisory role with one of the authors XZ.

## References

[B1] Adams T. D. Davidson L. E. Litwin S. E. Kim J. Kolotkin R. L. Nanjee M. N. (2017). Weight and metabolic outcomes 12 years after gastric bypass. *New Engl. J. Med.* 377 1143–1155. 10.1056/NEJMoa1700459 28930514 PMC5737957

[B2] Albaugh V. L. Banan B. Antoun J. Xiong Y. Guo Y. Ping J. (2018). Role of bile acids and GLP-1 in mediating the metabolic improvements of bariatric surgery. *Gastroenterology* 156 1041–1051.e4. 10.1053/j.gastro.2018.11.017 30445014 PMC6409186

[B3] Albaugh V. L. Flynn C. R. Cai S. Xiao Y. Tamboli R. A. Abumrad N. N. (2015). Early increases in bile acids post roux-en-Y gastric bypass are driven by insulin-sensitizing, secondary bile acids. *J. Clin. Endocrinol. Metab.* 100 E1225–E1233. 10.1210/jc.2015-2467 26196952 PMC4570157

[B4] Benedetti G. Mingrone G. Marcoccia S. Benedetti M. Giancaterini A. Greco A. V. (2000). Body composition and energy expenditure after weight loss following bariatric surgery. *J. Am. Coll. Nutr.* 19 270–274. 10.1080/07315724.2000.10718926 10763909

[B5] Bhutta H. Y. Rajpal N. White W. Freudenberg J. M. Liu Y. Way J. (2015). Effect of Roux-en-Y gastric bypass surgery on bile acid metabolism in normal and obese diabetic rats. *PLoS One* 10:e0122273. 10.1371/journal.pone.0122273 25798945 PMC4370587

[B6] Borg C. M. le Roux C. W. Ghatei M. A. Bloom S. R. Patel A. G. Aylwin S. J. (2006). Progressive rise in gut hormone levels after Roux-en-Y gastric bypass suggests gut adaptation and explains altered satiety. *Br. J. Surg.* 93 210–215. 10.1002/bjs.5227 16392104

[B7] Bozadjieva N. Heppner K. M. Seeley R. J. (2018). Targeting FXR and FGF19 to treat metabolic diseases-lessons learned from bariatric surgery. *Diabetes* 67 1720–1728. 10.2337/dbi17-0007 30135133 PMC6463577

[B8] Broeders E. P. Nascimento E. B. Havekes B. Brans B. Roumans K. H. Tailleux A. (2015). The bile acid chenodeoxycholic acid increases human brown adipose tissue activity. *Cell Metab.* 22 418–426. 10.1016/j.cmet.2015.07.002 26235421

[B9] Cariou B. van Harmelen K. Duran-Sandoval D. van Dijk T. H. Grefhorst A. Abdelkarim M. (2006). The farnesoid X receptor modulates adiposity and peripheral insulin sensitivity in mice. *J. Biol. Chem.* 281 11039–11049. 10.1074/jbc.M510258200 16446356

[B10] Carrasco F. Papapietro K. Csendes A. Salazar G. Echenique C. Lisboa C. (2007). Changes in resting energy expenditure and body composition after weight loss following Roux-en-Y gastric bypass. *Obes. Surg.* 17 608–616. 10.1007/s11695-007-9117-z 17658019

[B11] Cummings B. P. Bettaieb A. Graham J. L. Kim J. Ma F. Shibata N. (2013). Bile-acid-mediated decrease in endoplasmic reticulum stress: a potential contributor to the metabolic benefits of ileal interposition surgery in UCD-T2DM rats. *Dis. Model. Mech.* 6 443–456. 10.1242/dmm.010421 23264565 PMC3597026

[B12] Cummings B. P. Bettaieb A. Graham J. L. Stanhope K. L. Kowala M. Haj F. G. (2012). Vertical sleeve gastrectomy improves glucose and lipid metabolism and delays diabetes onset in UCD-T2DM rats. *Endocrinology* 153 3620–3632. 10.1210/en.2012-1131 22719048 PMC3404344

[B13] de Siqueira Cardinelli C. Torrinhas R. S. Sala P. Pudenzi M. A. Fernando F. A. C. Marques da Silva M. (2019). Fecal bile acid profile after Roux-en-Y gastric bypass and its association with the remission of type 2 diabetes in obese women: a preliminary study. *Clin. Nutr.* 10.1016/j.clnu.2018.12.028 [Epub ahead of print]. 30799193

[B14] Devkota S. Wang Y. Musch M. W. Leone V. Fehlner-Peach H. Nadimpalli A. (2012). Dietary-fat-induced taurocholic acid promotes pathobiont expansion and colitis in Il10-/- mice. *Nature* 487 104–108. 10.1038/nature11225 22722865 PMC3393783

[B15] Dicksved J. Lindberg M. Rosenquist M. Enroth H. Jansson J. K. Engstrand L. (2009). Molecular characterization of the stomach microbiota in patients with gastric cancer and in controls. *J. Med. Microbiol.* 58(Pt 4), 509–516. 10.1099/jmm.0.007302-0 19273648

[B16] Ermini M. Iaconis E. Mori A. (1991). The effects of bilio-jejunal diversion on streptozotocin diabetes in the rat. *Acta Diabetol. Lat.* 28 79–89. 10.1007/BF02732117 1862694

[B17] Fang S. Suh J. M. Reilly S. M. Yu E. Osborn O. Lackey D. (2015). Intestinal FXR agonism promotes adipose tissue browning and reduces obesity and insulin resistance. *Nat. Med.* 21 159–165. 10.1038/nm.3760 25559344 PMC4320010

[B18] Faria S. L. Faria O. P. Buffington C. de Almeida Cardeal M. Rodrigues de Gouvea H. (2012). Energy expenditure before and after Roux-en-Y gastric bypass. *Obes. Surg.* 22 1450–1455. 10.1007/s11695-012-0672-6 22592393

[B19] Ferrannini E. Camastra S. Astiarraga B. Nannipieri M. Castro-Perez J. Xie D. (2015). Increased bile acid synthesis and deconjugation after biliopancreatic diversion. *Diabetes* 64 3377–3385. 10.2337/db15-0214 26015549 PMC4587641

[B20] Flynn C. R. Albaugh V. L. Cai S. Cheung-Flynn J. Williams P. E. Brucker R. M. (2015). Bile diversion to the distal small intestine has comparable metabolic benefits to bariatric surgery. *Nat. Commun.* 6:7715. 10.1038/ncomms8715 26197299 PMC4518285

[B21] Furet J. P. Kong L. C. Tap J. Poitou C. Basdevant A. Bouillot J. L. (2010). Differential adaptation of human gut microbiota to bariatric surgery-induced weight loss: links with metabolic and low-grade inflammation markers. *Diabetes* 59 3049–3057. 10.2337/db10-0253 20876719 PMC2992765

[B22] Gerhard G. S. Styer A. M. Wood G. C. Roesch S. L. Petrick A. T. Gabrielsen J. (2013). A role for fibroblast growth factor 19 and bile acids in diabetes remission after Roux-en-Y gastric bypass. *Diabetes Care* 36 1859–1864. 10.2337/dc12-2255 23801799 PMC3687273

[B23] Goncalves D. Barataud A. De Vadder F. Vinera J. Zitoun C. Duchampt A. (2015). Bile routing modification reproduces key features of gastric bypass in rat. *Ann. Surg.* 262 1006–1015. 10.1097/SLA.0000000000001121 25575265 PMC5678047

[B24] Goodwin B. Jones S. A. Price R. R. Watson M. A. McKee D. D. Moore L. B. (2000). A regulatory cascade of the nuclear receptors FXR, SHP-1, and LRH-1 represses bile acid biosynthesis. *Mol. Cell* 6 517–526. 10.1016/S1097-2765(00)00051-4 11030332

[B25] Graessler J. Qin Y. Zhong H. Zhang J. Licinio J. Wong M. L. (2013). Metagenomic sequencing of the human gut microbiome before and after bariatric surgery in obese patients with type 2 diabetes: correlation with inflammatory and metabolic parameters. *Pharmacogenomics J* 13 514–522. 10.1038/tpj.2012.43 23032991

[B26] Haluzikova D. Lacinova Z. Kavalkova P. Drapalova J. Krizova J. Bartlova M. (2013). Laparoscopic sleeve gastrectomy differentially affects serum concentrations of FGF-19 and FGF-21 in morbidly obese subjects. *Obesity* 21 1335–1342. 10.1002/oby.20208 23670968

[B27] Han H. Wang L. Du H. Jiang J. Hu C. Zhang G. (2015). Expedited biliopancreatic juice flow to the distal gut benefits the diabetes control after duodenal-jejunal bypass. *Obes. Surg.* 25 1802–1809. 10.1007/s11695-015-1633-7 25726319

[B28] Inagaki T. Choi M. Moschetta A. Peng L. Cummins C. L. McDonald J. G. (2005). Fibroblast growth factor 15 functions as an enterohepatic signal to regulate bile acid homeostasis. *Cell Metab.* 2 217–225. 10.1016/j.cmet.2005.09.001 16213224

[B29] Inagaki T. Moschetta A. Lee Y. K. Peng L. Zhao G. Downes M. (2006). Regulation of antibacterial defense in the small intestine by the nuclear bile acid receptor. *Proc. Natl. Acad. Sci. U.S.A.* 103 3920–3925. 10.1073/pnas.0509592103 16473946 PMC1450165

[B30] Jahansouz C. Staley C. Bernlohr D. A. Sadowsky M. J. Khoruts A. Ikramuddin S. (2017). Sleeve gastrectomy drives persistent shifts in the gut microbiome. *Surg. Obes. Relat. Dis.* 13 916–924. 10.1016/j.soard.2017.01.003 28279578

[B31] Jansen P. L. van Werven J. Aarts E. Berends F. Janssen I. Stoker J. (2011). Alterations of hormonally active fibroblast growth factors after Roux-en-Y gastric bypass surgery. *Dig. Dis.* 29 48–51. 10.1159/000324128 21691104

[B32] Jiang C. Xie C. Lv Y. Li J. Krausz K. W. Shi J. (2015). Intestine-selective farnesoid X receptor inhibition improves obesity-related metabolic dysfunction. *Nat. Commun.* 6:10166. 10.1038/ncomms10166 26670557 PMC4682112

[B33] Jorgensen N. B. Dirksen C. Bojsen-Moller K. N. Kristiansen V. B. Wulff B. S. Rainteau D. (2015). Improvements in glucose metabolism early after gastric bypass surgery are not explained by increases in total bile acids and fibroblast growth factor 19 concentrations. *J. Clin. Endocrinol. Metab.* 100 E396–E406. 10.1210/jc.2014-1658 25536209

[B34] Kindel T. L. Krause C. Helm M. C. McBride C. L. Oleynikov D. Thakare R. (2018). Increased glycine-amidated hyocholic acid correlates to improved early weight loss after sleeve gastrectomy. *Surg. Endosc.* 32 805–812. 10.1007/s00464-017-5747-y 28779240 PMC5844265

[B35] Kir S. Beddow S. A. Samuel V. T. Miller P. Previs S. F. Suino-Powell K. (2011). FGF19 as a postprandial, insulin-independent activator of hepatic protein and glycogen synthesis. *Science* 331 1621–1624. 10.1126/science.1198363 21436455 PMC3076083

[B36] Kohli R. Bradley D. Setchell K. D. Eagon J. C. Abumrad N. Klein S. (2013a). Weight loss induced by Roux-en-Y gastric bypass but not laparoscopic adjustable gastric banding increases circulating bile acids. *J. Clin. Endocrinol. Metab.* 98 E708–E712. 10.1210/jc.2012-3736 23457410 PMC3615197

[B37] Kohli R. Setchell K. D. Kirby M. Myronovych A. Ryan K. K. Ibrahim S. H. (2013b). A surgical model in male obese rats uncovers protective effects of bile acids post-bariatric surgery. *Endocrinology* 154 2341–2351. 10.1210/en.2012-2069 23592746 PMC3689286

[B38] Laferrere B. Heshka S. Wang K. Khan Y. McGinty J. Teixeira J. (2007). Incretin levels and effect are markedly enhanced 1 month after Roux-en-Y gastric bypass surgery in obese patients with type 2 diabetes. *Diabetes Care* 30 1709–1716. 10.2337/dc06-1549 17416796 PMC2743330

[B39] Larsen N. Vogensen F. K. van den Berg F. W. Nielsen D. S. Andreasen A. S. Pedersen B. K. (2010). Gut microbiota in human adults with type 2 diabetes differs from non-diabetic adults. *PLoS One* 5:e9085. 10.1371/journal.pone.0009085 20140211 PMC2816710

[B40] le Roux C. W. Welbourn R. Werling M. Osborne A. Kokkinos A. Laurenius A. (2007). Gut hormones as mediators of appetite and weight loss after Roux-en-Y gastric bypass. *Ann. Surg.* 246 780–785. 10.1097/SLA.0b013e3180caa3e3 17968169

[B41] Ley R. E. Turnbaugh P. J. Klein S. Gordon J. I. (2006). Microbial ecology: human gut microbes associated with obesity. *Nature* 444 1022–1023. 10.1038/4441022a 17183309

[B42] Li J. V. Ashrafian H. Bueter M. Kinross J. Sands C. le Roux C. W. (2011). Metabolic surgery profoundly influences gut microbial-host metabolic cross-talk. *Gut* 60 1214–1223. 10.1136/gut.2010.234708 21572120 PMC3677150

[B43] Lu T. T. Makishima M. Repa J. J. Schoonjans K. Kerr T. A. Auwerx J. (2000). Molecular basis for feedback regulation of bile acid synthesis by nuclear receptors. *Mol. Cell* 6 507–515. 10.1016/S1097-2765(00)00050-2 11030331

[B44] Ma K. Saha P. K. Chan L. Moore D. D. (2006). Farnesoid X receptor is essential for normal glucose homeostasis. *J. Clin. Invest.* 116 1102–1109. 10.1172/JCI25604 16557297 PMC1409738

[B45] Madsbad S. Holst J. J. (2014). GLP-1 as a mediator in the remission of type 2 diabetes after gastric bypass and sleeve gastrectomy surgery. *Diabetes* 63 3172–3174. 10.2337/db14-0935 25249642

[B46] Makishima M. Okamoto A. Y. Repa J. J. Tu H. Learned R. M. Luk A. (1999). Identification of a nuclear receptor for bile acids. *Science* 284 1362–1365. 10.1126/science.284.5418.1362 10334992

[B47] Manfredini G. Ermini M. Scopsi L. Bonaguidi F. Ferrannini E. (1985). Internal biliary diversion improves glucose tolerance in the rat. *Am. J. Physiol.* 249(4 Pt 1), G519–G527. 10.1152/ajpgi.1985.249.4.G519 3901778

[B48] Mans E. Serra-Prat M. Palomera E. Sunol X. Clave P. (2015). Sleeve gastrectomy effects on hunger, satiation, and gastrointestinal hormone and motility responses after a liquid meal test. *Am. J. Clin. Nutr.* 102 540–547. 10.3945/ajcn.114.104307 26201818

[B49] Maruyama T. Miyamoto Y. Nakamura T. Tamai Y. Okada H. Sugiyama E. (2002). Identification of membrane-type receptor for bile acids (M-BAR). *Biochem. Biophys. Res. Commun.* 298 714–719. 10.1016/S0006-291X(02)02550-0 12419312

[B50] McGavigan A. K. Garibay D. Henseler Z. M. Chen J. Bettaieb A. Haj F. G. (2017). TGR5 contributes to glucoregulatory improvements after vertical sleeve gastrectomy in mice. *Gut* 66 226–234. 10.1136/gutjnl-2015-309871 26511794 PMC5512436

[B51] Mika A. Kaska L. Proczko-Stepaniak M. Chomiczewska A. Swierczynski J. Smolenski R. T. (2018). Evidence that the length of bile loop determines serum bile acid concentration and glycemic control after bariatric surgery. *Obes. Surg.* 28 3405–3414. 10.1007/s11695-018-3314-9 29790128

[B52] Miyachi T. Nagao M. Shibata C. Kitahara Y. Tanaka N. Watanabe K. (2016). Biliopancreatic limb plays an important role in metabolic improvement after duodenal-jejunal bypass in a rat model of diabetes. *Surgery* 159 1360–1371. 10.1016/j.surg.2015.11.027 26767308

[B53] Mueller M. Thorell A. Claudel T. Jha P. Koefeler H. Lackner C. (2015). Ursodeoxycholic acid exerts farnesoid X receptor-antagonistic effects on bile acid and lipid metabolism in morbid obesity. *J. Hepatol.* 62 1398–1404. 10.1016/j.jhep.2014.12.034 25617503 PMC4451470

[B54] Murphy R. Tsai P. Jullig M. Liu A. Plank L. Booth M. (2017). Differential changes in gut microbiota after gastric bypass and sleeve gastrectomy bariatric surgery vary according to diabetes remission. *Obes. Surg.* 27 917–925. 10.1007/s11695-016-2399-2 27738970

[B55] Myronovych A. Kirby M. Ryan K. K. Zhang W. Jha P. Setchell K. D. (2014a). Vertical sleeve gastrectomy reduces hepatic steatosis while increasing serum bile acids in a weight-loss-independent manner. *Obesity* 22 390–400. 10.1002/oby.20548 23804416 PMC3836901

[B56] Myronovych A. Salazar-Gonzalez R. M. Ryan K. K. Miles L. Zhang W. Jha P. (2014b). The role of small heterodimer partner in nonalcoholic fatty liver disease improvement after sleeve gastrectomy in mice. *Obesity* 22 2301–2311. 10.1002/oby.20890 25376397 PMC4286402

[B57] Nakatani H. Kasama K. Oshiro T. Watanabe M. Hirose H. Itoh H. (2009). Serum bile acid along with plasma incretins and serum high-molecular weight adiponectin levels are increased after bariatric surgery. *Metabolism* 58 1400–1407. 10.1016/j.metabol.2009.05.006 19570554

[B58] Nauck M. A. Homberger E. Siegel E. G. Allen R. C. Eaton R. P. Ebert R. (1986). Incretin effects of increasing glucose loads in man calculated from venous insulin and C-peptide responses. *J. Clin. Endocrinol. Metab.* 63 492–498. 10.1210/jcem-63-2-492 3522621

[B59] Noh D. O. Gilliland S. E. (1993). Influence of bile on cellular integrity and beta-galactosidase activity of *Lactobacillus acidophilus*. *J. Dairy Sci.* 76 1253–1259. 10.3168/jds.S0022-0302(93)77454-8 8505417

[B60] Parks D. J. Blanchard S. G. Bledsoe R. K. Chandra G. Consler T. G. Kliewer S. A. (1999). Bile acids: natural ligands for an orphan nuclear receptor. *Science* 284 1365–1368. 10.1126/science.284.5418.1365 10334993

[B61] Pournaras D. J. Glicksman C. Vincent R. P. Kuganolipava S. Alaghband-Zadeh J. Mahon D. (2012). The role of bile after Roux-en-Y gastric bypass in promoting weight loss and improving glycaemic control. *Endocrinology* 153 3613–3619. 10.1210/en.2011-2145 22673227 PMC3404349

[B62] Prakash S. Tomaro-Duchesneau C. Saha S. Cantor A. (2011). The gut microbiota and human health with an emphasis on the use of microencapsulated bacterial cells. *J. Biomed. Biotechnol.* 2011:981214. 10.1155/2011/981214 21772792 PMC3134400

[B63] Qin J. Li Y. Cai Z. Li S. Zhu J. Zhang F. (2012). A metagenome-wide association study of gut microbiota in type 2 diabetes. *Nature* 490 55–60. 10.1038/nature11450 23023125

[B64] Rabl C. Rao M. N. Schwarz J. M. Mulligan K. Campos G. M. (2014). Thermogenic changes after gastric bypass, adjustable gastric banding or diet alone. *Surgery* 156 806–812. 10.1016/j.surg.2014.06.070 25239323 PMC4171693

[B65] Ridlon J. M. Kang D. J. Hylemon P. B. (2006). Bile salt biotransformations by human intestinal bacteria. *J. Lipid Res.* 47 241–259. 10.1194/jlr.R500013-JLR200 16299351

[B66] Ryan K. K. Tremaroli V. Clemmensen C. Kovatcheva-Datchary P. Myronovych A. Karns R. (2014). FXR is a molecular target for the effects of vertical sleeve gastrectomy. *Nature* 509 183–188. 10.1038/nature13135 24670636 PMC4016120

[B67] Sachdev S. Wang Q. Billington C. Connett J. Ahmed L. Inabnet W. (2016). FGF 19 and bile acids increase following Roux-en-Y gastric bypass but not after medical management in patients with type 2 diabetes. *Obes. Surg.* 26 957–965. 10.1007/s11695-015-1834-0 26259981 PMC4751075

[B68] Schauer P. R. Bhatt D. L. Kirwan J. P. Wolski K. Aminian A. Brethauer S. A. (2017). Bariatric surgery versus intensive medical therapy for diabetes - 5-year outcomes. *New Engl. J. Med.* 376 641–651. 10.1056/NEJMoa1600869 28199805 PMC5451258

[B69] Scopinaro N. (2006). Biliopancreatic diversion: mechanisms of action and long-term results. *Obes. Surg.* 16 683–689. 10.1381/096089206777346637 16756725

[B70] Shao Y. Shen Q. Hua R. Evers S. S. He K. Yao Q. (2018). Effects of sleeve gastrectomy on the composition and diurnal oscillation of gut microbiota related to the metabolic improvements. *Surg. Obes. Relat. Dis.* 14 731–739. 10.1016/j.soard.2018.02.024 29680673

[B71] Sinal C. J. Tohkin M. Miyata M. Ward J. M. Lambert G. Gonzalez F. J. (2000). Targeted disruption of the nuclear receptor FXR/BAR impairs bile acid and lipid homeostasis. *Cell* 102 731–744. 10.1016/S0092-8674(00)00062-3 11030617

[B72] Sips F. L. P. Eggink H. M. Hilbers P. A. J. Soeters M. R. Groen A. K. van Riel N. A. W. (2018). Analysis identifies intestinal transit as a key determinant of systemic bile acid metabolism. *Front. Physiol.* 9:631. 10.3389/fphys.2018.00631 29951001 PMC6008656

[B73] Steinert R. E. Peterli R. Keller S. Meyer-Gerspach A. C. Drewe J. Peters T. (2013). Bile acids and gut peptide secretion after bariatric surgery: a 1-year prospective randomized pilot trial. *Obesity* 21 E660–E668. 10.1002/oby.20522 23804517

[B74] Tacchino R. M. Mancini A. Perrelli M. Bianchi A. Giampietro A. Milardi D. (2003). Body composition and energy expenditure: relationship and changes in obese subjects before and after biliopancreatic diversion. *Metabolism* 52 552–558. 10.1053/meta.2003.50109 12759883

[B75] Thomas C. Gioiello A. Noriega L. Strehle A. Oury J. Rizzo G. (2009). TGR5-mediated bile acid sensing controls glucose homeostasis. *Cell Metab.* 10 167–177. 10.1016/j.cmet.2009.08.001 19723493 PMC2739652

[B76] Tilg H. Kaser A. (2011). Gut microbiome, obesity, and metabolic dysfunction. *J. Clin. Investig.* 121 2126–2132. 10.1172/JCI58109 21633181 PMC3104783

[B77] Trabelsi M. S. Daoudi M. Prawitt J. Ducastel S. Touche V. Sayin S. I. (2015). Farnesoid X receptor inhibits glucagon-like peptide-1 production by enteroendocrine L cells. *Nat. Commun.* 6:7629. 10.1038/ncomms8629 26134028 PMC4579574

[B78] Tremaroli V. Karlsson F. Werling M. Stahlman M. Kovatcheva-Datchary P. Olbers T. (2015). Roux-en-Y gastric bypass and vertical banded gastroplasty induce long-term changes on the human gut microbiome contributing to fat mass regulation. *Cell Metab.* 22 228–238. 10.1016/j.cmet.2015.07.009 26244932 PMC4537510

[B79] Turnbaugh P. J. Hamady M. Yatsunenko T. Cantarel B. L. Duncan A. Ley R. E. (2009). A core gut microbiome in obese and lean twins. *Nature* 457 480–484. 10.1038/nature07540 19043404 PMC2677729

[B80] Turnbaugh P. J. Ley R. E. Hamady M. Fraser-Liggett C. M. Knight R. Gordon J. I. (2007). The human microbiome project. *Nature* 449 804–810. 10.1038/nature06244 17943116 PMC3709439

[B81] Turnbaugh P. J. Ley R. E. Mahowald M. A. Magrini V. Mardis E. R. Gordon J. I. (2006). An obesity-associated gut microbiome with increased capacity for energy harvest. *Nature* 444 1027–1031. 10.1038/nature05414 17183312

[B82] Vrieze A. Van Nood E. Holleman F. Salojarvi J. Kootte R. S. Bartelsman J. F. (2012). Transfer of intestinal microbiota from lean donors increases insulin sensitivity in individuals with metabolic syndrome. *Gastroenterology* 143 913–6e7. 10.1053/j.gastro.2012.06.031 22728514

[B83] Wang H. Chen J. Hollister K. Sowers L. C. Forman B. M. (1999). Endogenous bile acids are ligands for the nuclear receptor FXR/BAR. *Mol. Cell* 3 543–553. 10.1016/S1097-2765(00)80348-2 10360171

[B84] Watanabe M. Houten S. M. Mataki C. Christoffolete M. A. Kim B. W. Sato H. (2006). Bile acids induce energy expenditure by promoting intracellular thyroid hormone activation. *Nature* 439 484–489. 10.1038/nature04330 16400329

[B85] Watanabe M. Houten S. M. Wang L. Moschetta A. Mangelsdorf D. J. Heyman R. A. (2004). Bile acids lower triglyceride levels via a pathway involving FXR, SHP, and SREBP-1c. *J. Clin. Invest.* 113 1408–1418. 10.1172/JCI21025 15146238 PMC406532

[B86] Wu T. Bound M. J. Standfield S. D. Gedulin B. Jones K. L. Horowitz M. (2013). Effects of rectal administration of taurocholic acid on glucagon-like peptide-1 and peptide YY secretion in healthy humans. *Diabetes Obes. Metab.* 15 474–477. 10.1111/dom.12043 23181598

[B87] Wu T. Ma J. Bound M. J. Checklin H. Deacon C. F. Jones K. L. (2014). Effects of sitagliptin on glycemia, incretin hormones, and antropyloroduodenal motility in response to intraduodenal glucose infusion in healthy lean and obese humans and patients with type 2 diabetes treated with or without metformin. *Diabetes* 63 2776–2787. 10.2337/db13-1627 24647737

[B88] Wu T. Rayner C. K. Horowitz M. (2016). Incretins. *Handb. Exp. Pharmacol.* 233 137–171. 10.1007/164_2015_9 25903418

[B89] Zhang H. DiBaise J. K. Zuccolo A. Kudrna D. Braidotti M. Yu Y. (2009). Human gut microbiota in obesity and after gastric bypass. *Proc. Natl. Acad. Sci. U.S.A.* 106 2365–2370. 10.1073/pnas.0812600106 19164560 PMC2629490

[B90] Zhang X. Liu T. Wang Y. Zhong M. Zhang G. Liu S. (2016). Comparative effects of bile diversion and duodenal-jejunal bypass on glucose and lipid metabolism in male diabetic rats. *Obes. Surg.* 26 1565–1575. 10.1007/s11695-015-1925-y 26464241

[B91] Zhang X. Young R. L. Bound M. Hu S. Jones K. L. Horowitz M. (2019). Comparative effects of proximal and distal small intestinal glucose exposure on glycemia, incretin hormone secretion, and the incretin effect in health and type 2 diabetes. *Diabetes Care* 42 520–528. 10.2337/dc18-2156 30765429

[B92] Zhang Y. Lee F. Y. Barrera G. Lee H. Vales C. Gonzalez F. J. (2006). Activation of the nuclear receptor FXR improves hyperglycemia and hyperlipidemia in diabetic mice. *Proc. Natl. Acad. Sci. U.S.A.* 103 1006–1011. 10.1073/pnas.0506982103 16410358 PMC1347977

[B93] Zhernakova A. Kurilshikov A. Bonder M. J. Tigchelaar E. F. Schirmer M. Vatanen T. (2016). Population-based metagenomics analysis reveals markers for gut microbiome composition and diversity. *Science* 352 565–569. 10.1126/science.aad3369 27126040 PMC5240844

